# Split selectable marker systems utilizing inteins facilitate gene stacking in plants

**DOI:** 10.1038/s42003-023-04950-8

**Published:** 2023-05-26

**Authors:** Guoliang Yuan, Haiwei Lu, Kuntal De, Md Mahmudul Hassan, Yang Liu, Md. Torikul Islam, Wellington Muchero, Gerald A. Tuskan, Xiaohan Yang

**Affiliations:** 1 Biosciences Division, Oak Ridge National Laboratory, Oak Ridge, TN 37831 USA; 2 The Center for Bioenergy Innovation, Oak Ridge National Laboratory, Oak Ridge, TN 37831 USA; 3 Chemical and Biological Process Development Group, Pacific Northwest National Laboratory, 902 Battelle Boulevard, Richland, WA 99352 USA; 4 Department of Academic Education, Central Community College—Hastings, Hastings, NE 68902 USA; 5 Department of Genetics and Plant Breeding, Patuakhali Science and Technology University, Dumki, Patuakhali, 8602 Bangladesh

**Keywords:** Genetic vectors, Gene expression, Molecular engineering in plants, Molecular engineering in plants

## Abstract

The ability to stack multiple genes in plants is of great importance in the development of crops with desirable traits but can be challenging due to limited selectable marker options. Here we establish split selectable marker systems using protein splicing elements called “inteins” for *Agrobacterium*-mediated co-transformation in plants. First, we show that such a split selectable marker system can be used effectively in plants to reconstitute a visible marker, RUBY, from two non-functional fragments through tobacco leaf infiltration. Next, to determine the general applicability of our split selectable marker systems, we demonstrate the utility of these systems in the model plants *Arabidopsis* and poplar by successfully stacking two reporters *eYGFPuv* and *RUBY*, using split Kanamycin or Hygromycin resistance markers. In conclusion, this method enables robust plant co-transformation, providing a valuable tool for the simultaneous insertion of multiple genes into both herbaceous and woody plants efficiently.

## Abstract (web summary)

Establishment of multiple split selectable marker systems that utilize inteins to enable selection of transgenic plants stably transformed with two T-DNAs via *Agrobacterium*-mediated transformation.

## Introduction

Metabolic engineering in plants relies on the introduction of complete or partial synthetic pathways into the target plant to create novel plant traits and produce value-added metabolites and therapeutic proteins^1,2^. A complete synthetic pathway is typically encoded by multiple genes that involve multiple genetic parts and gene circuits. Multigene engineering, therefore, is becoming more and more important for plant synthetic biology research. Also, a lot of complex plant traits (e.g., yield) are controlled by multiple genes, and as such, genetic improvement of such polygenic traits requires multi-gene stacking. *Agrobacterium*-mediated transformation is, to date, the most widely used method for plant genetic engineering due to its relatively high efficiency^3^. Although some progress in *Agrobacterium*-mediated transformation of large DNA fragments required for multigene engineering in plants has been achieved, it has been reported that large genomic DNA fragments are not stable in *Agrobacterium*, and T-DNA can be truncated at the left and/or right ends before being inserted into the plant genome^4–6^. Thus, the effective transformation of tens of genes into a plant genome and consequent optimal control of gene expression remain unattainable in plant engineering. Here, we developed a split-intein-based gene-stacking method through split-selectable-marker-enabled co-transformation in *Arabidopsis thaliana* and poplar (*Populus*).

**Fig. 1:**
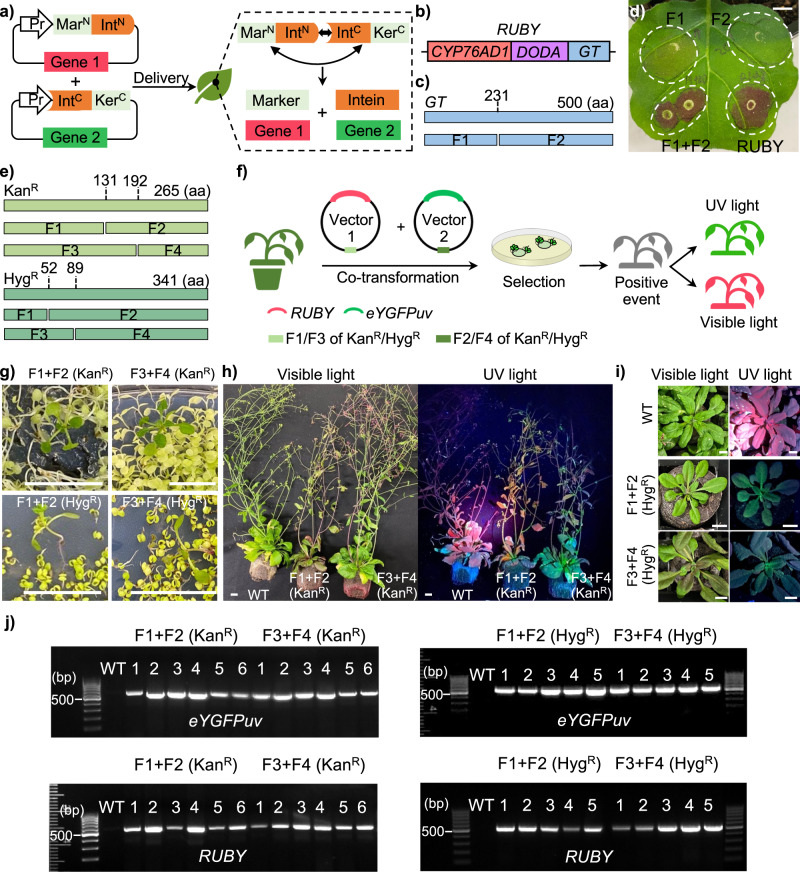
Split RUBY and the split–selectable markers mediated gene stacking in tobacco and *Arabidopsis*. **a** Split selectable marker for co-selection of two separate transgenic vectors. **b** Illustration of *RUBY* reporter. **c** Identification of the potential split site of gene *GT*. **d** Transient expression of split-*RUBY* in *N. benthamiana*. Scale bar, 1 cm. **e** Identification of potential split sites of gene *Kan*^*R*^ and *Hyg*^*R*^. **f** Experiment design of the split–selectable markers mediated co-transformation in plants. **g** Selection of T1 seedling on MS medium containing Kanamycin or Hygromycin. Scale bar, 1 cm. **h** Phenotyping of Kanamycin resistant T1 transformants (6 weeks). Scale bar, 1 cm. **i** Phenotyping of Hygromycin resistant T1 transformants (four weeks). Scale bar, 1 cm. **j** Genotyping of T1 transformants using primers of *eYGFPuv* and *RUBY*, respectively.

An intein is an intervening protein domain that excises itself post-translationally from the host protein leading to the ligation of flanking N- and C-terminal residues (termed as exteins), which share a common intein, to form a new protein (Fig. 1a)^7^. Split inteins to date have enabled the development of numerous tools for both synthetic and biological applications by providing a rapid and bioorthogonal means to link two polypeptides^7,8^. As such, we previously showed that split intein, derived from *Npu*DnaE^9^, can be used to reduce the size of the CRISPR/Cas9 system by splitting Cas9 into multiple fragments, leading to effective base editing in plants^10^. In this study, we aim to develop split selectable markers using split inteins to enable single-selectable-marker-gene dependent co-transformation in plants.

## Results

### Intein-mediated split *RUBY* reporter in tobacco leaf infiltration

Initially, we selected *eYGFPuv*^11^ and *RUBY*^12^ as the reporter genes that can be easily visualized by naked eyes with and without UV light, respectively, to establish a functional split system. In fact, the *RUBY* reporter is encoded by three genes *CYP76AD1*, *DODA*, and *glucosyltransferase* (*GT*) (Fig. 1b). In general, a catalytic Cys residue at position +1 of the C-terminal extein is mandatory to maintain substantial splicing activities^7^. A potential split site, L231:C232, was thus identified for splitting the gene *GT* into two fragments, termed GTf1 and GTf2 (Fig. 1c). We split the *RUBY* reporter into two parts by creating plasmid pAXY0006 containing *CYP76AD1*, *DODA* and GTf1-NpuDnaE(N), and plasmid pAXY0007 containing NpuDnaE(C)-GTf2 (Supplementary Fig. 1). Note that the *Arabidopsis* codon-optimized *Npu*DnaE intein was created to improve gene expression and translational efficiency in plants. We then tested split-*RUBY* using *Agrobacterium*-mediated leaf infiltration in *Nicotiana benthamiana*. The strong red pigment was observed by naked eyes both in the positive control (RUBY) and the leaf area co-infiltrated with pAXY0006 and pAXY0007 plasmids, whereas no red pigment was detected in the leaf area infiltrated with pAXY0006 or pAXY0007 plasmids alone (negative controls) (Fig. 1d). Taken together, our results indicate that the functional RUBY reporter was restored by split inteins, which is consistent with the split-*eYGFPuv* reported previously^10^.

### The intein-mediated split selectable marker in *Arabidopsis*

Because Kanamycin resistance (Kan^R^; *nptII*) and Hygromycin resistance (Hyg^R^; *hpt*) are widely used as the selectable markers in plant transformation, we next tested *Npu*DnaE intein^9^ for splitting the *nptII* gene encoding neomycin phosphotransferase II and the *hpt* gene encoding Hygromycin phosphotransferase, which confers Kan^R^ and Hyg^R^, respectively. Following the rule of obligatory cysteine residue on the C-extein^7^, we identified two split sites (T131:C132 and A192:C193) for *nptII* and two split sites (S52:C53 and Y89:C90) for *hpt* (Fig. 1e).

The coding sequence of *nptII* or *hpt* was split into an N-terminal fragment (Mar^N^, F1/F3) and a C-terminal fragment (Ker^C^, F2/F4), which were then cloned upstream of an N-terminal fragment of the *Npu*DnaE intein (Int^N^) and downstream of a C-terminal fragment of the *Npu*DnaE intein (Int^C^), respectively, into two vectors (Fig. 1a, e). Each vector also carries one of the two reporters (*eYGFPuv* and *RUBY*), which allows for easy assessment of co-transformation (Supplementary Fig. 2). Thus, we expect to see both green fluorescences under UV light and red pigment under visible light in Kanamycin-resistant or Hygromycin-resistant transgenic plants after co-transformation of these two vectors (Fig. 1f).

**Fig. 2:**
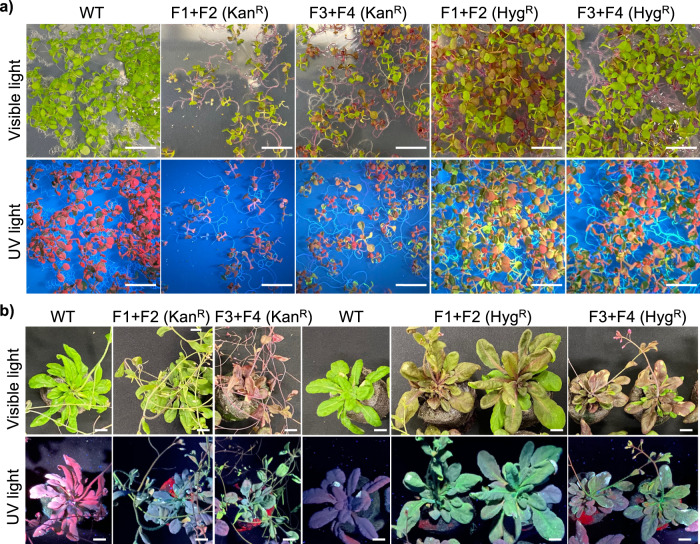
Analysis of T2 plants after the split–selectable markers mediated gene stacking in *Arabidopsis*. **a** Selection of T2 seedlings on MS medium containing Kanamycin or Hygromycin. **b** Phenotyping of Kanamycin-resistant T2 transformants (5 weeks) and Hygromycin-resistant T2 transformants (4 weeks). Scale bar, 1 cm.

After co-transformation via a floral dip in *Arabidopsis*, multiple transgenic seedlings with typical Kanamycin-resistant or Hygromycin-resistant phenotype were successfully identified on the selection media, indicating that the two inactive fragments of each selectable marker gene (*nptII* or *hpt*) were effectively reconstituted post-translationally (Fig. 1g). Subsequently, the green fluorescence and red pigment were observed at different stages of Kanamycin-resistant T1 plants (Fig. 1h) and Hygromycin-resistant T1 plants (Fig. 1i) under UV light and visible light, respectively, suggesting that both eYGFPuv and RUBY vectors were also transformed into the same plant simultaneously through split-Kan^R^- or split-Hyg^R^-mediated co-transformation. These observations were further confirmed by polymerase chain reaction (PCR)-based genotyping, where both *eYGFPuv* and *RUBY* genes were detected in all Kanamycin-resistant or Hygromycin-resistant plants (Fig. 1j and Supplementary Fig. 3). Next, we evaluated the phenotype of T2 generations of above transgenic plants, with the expectation that the Kanamycin-resistance or Hygromycin-resistance phenotype will be observed in T2 seedlings, along with green fluorescence under UV light and red pigment under visible light, as seen in Fig. 2a. Due to segregation of T-DNA inserts in the offspring, not all T2 plants exhibit the phenotype of antibiotic resistance and expression of both eYGFPuv and RUBY, but the phenotype of eYGFPuv and RUBY was observed in most resistant T2 plants though the expression level varies among plants (Fig. 2a). The phenotypes of eYGFPuv and RUBY were continuously detected in the mature plants of Kanamycin-resistant lines and Hygromycin-resistant lines (Fig. 2b and Supplementary Fig. 4). These findings were also supported by PCR-based genotyping, which revealed the presence of the *eYGFPuv* and *RUBY* genes in every plant that was either kanamycin- or hygromycin-resistant (Supplementary Fig. 5). Taken together, the traits of Kanamycin-resistant, Hygromycin-resistant, eYGFPuv and RUBY are all heritable in *Arabidopsis* across generations, demonstrating the split–Kan^R^**-** and split–Hyg^R^-mediated co-transformation are effective and robust methods for stable gene-stacking in plants.

### The intein-mediated split selectable marker in poplar

**Fig. 3:**
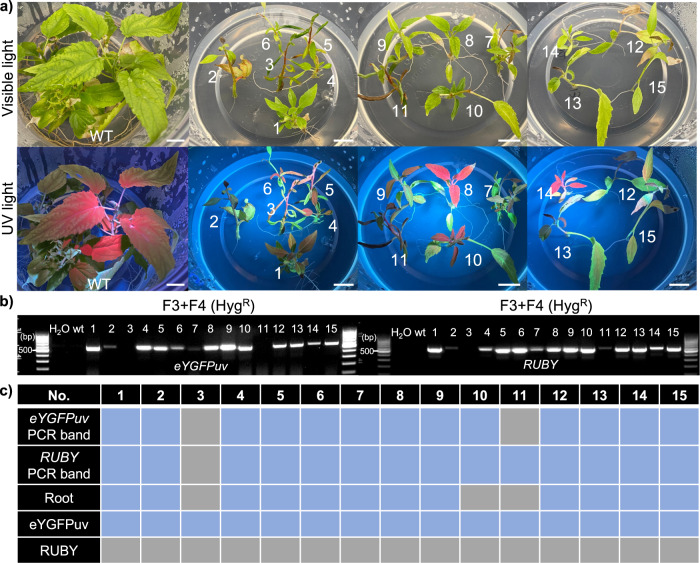
The split–selectable markers mediated gene stacking in poplar. **a** Root induction in root induction medium supplied with Hygromycin and phenotyping of transgenic events with/without UV light. Scale bar, 1 cm. **b** Genotyping of transgenic poplar events using primers of *eYGFPuv* and *RUBY*, respectively. **c** The analysis and alignment of genotyping and phenotyping of transgenic plants. The blue block represents positive events, while the gray block represents negative events.

We further examined the efficacy of split–Hyg^R^ system in poplar using vector pairs F3 and F4. After tissue-culture-based co-transformation in Poplar ‘717’ (*Populus tremula* x *alba* clone INRA ‘717-1B4’), we observed more than 20 transgenic shoots that showed bright green fluorescence under UV light. We randomly selected 15 eYGFPuv-expressing shoots and cultured them on a root induction medium supplied with Hygromycin (Fig. 3a), where 80% of induced shoots were rooted successfully on the selection medium, suggesting that functional Hygromycin phosphotransferase was generated post-translationally. We observed consistent green fluorescence in all rooted plants over time though some plants showed weaker and nonuniform eYGFPuv signals (Fig. 3a). Interestingly, typical RUBY phenotype was not observed in the rooted plants though red pigmentation was observed in the stem of some plants. Based on the RUBY expression in transgenic poplar plants generated previously^13^, red pigment is typical to be visible in different organs, including leaf, stem, and root. However, the red pigmentation in the stem of some plants can also be caused by stress, which is not rare during plant transformation. Intriguingly, we detected both *eYGFPuv* and *RUBY* genes via PCR genotyping in all rooted plants (Fig. 3b, c and Supplementary Fig. 6). Overall, these results suggest that the split–Hyg^R^ system can also work effectively in tissue-culture-based transformation in woody plants.

### Western blot analysis of protein trans-splicing of Hygromycin markertrons

**Fig. 4:**
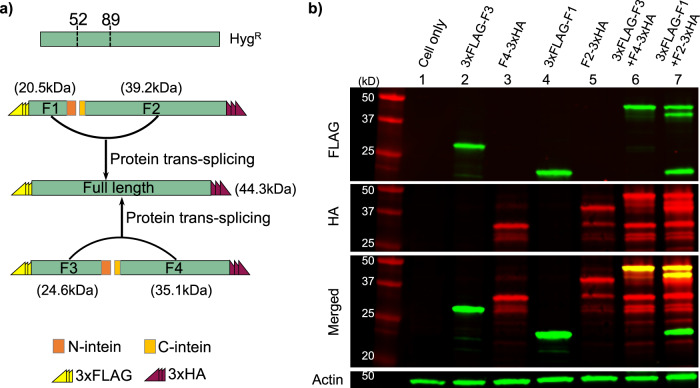
Western blot analysis of trans-splicing of the Hyg^R^ protein. **a** The N-terminal fragments of Hyg^R^ (F1 and F3) are N-terminally tagged with 3xFLAG epitope while the C-terminal fragments of Hyg^R^ (F2 and F4) are C-terminally tagged with 3xHA epitope. **b** Western blot was performed with the proteins extracted from human kidney cells, which were either transfected with the plasmids containing one of the fragments F1–F4, respectively, or co-transfected with F1 + F2 containing plasmids and F3 + F4 containing plasmids. Red, green, and yellow bands indicate FLAG, HA, and merged bands, respectively. Actin serves as the equal-loading control. Note: Fig. 4b shows cropped, contrast-adjusted image. Please see the original images of Fig. 4b in Supplementary Fig. 8.

To directly observe protein splicing and to confirm these inteins are indeed orthogonal, we conducted western blot analysis of protein trans-splicing between N-Hyg^R^ N-terminally tagged with 3xFLAG-epitope and C-Hyg^R^ C-terminally tagged with 3xHA-epitope (Fig. 4 and Supplementary Figure 7). As expected, full-length Hyg^R^ (lanes 6 and 7) was observed in the co-transformation of cognate Hyg^R^ fragments with matching N- and C-inteins while transformation with fragment F1/F2/F3/F4 only (lanes 2–5) did not yield full-length Hyg^R^ (Fig. 4b and Supplementary Fig. 8).

## Discussion

Current plant co-transformation approaches rely on at least two selectable gene markers. For this protocol, the concentrations of combined antibiotics need to be tested and adjusted carefully to achieve optimal transgenic selection effect. There is also a difference in selection efficacy between different selectable markers, such that, Hyg^R^ works better (lower rate of false positives) than Kan^R^ in the genetic transformation of some poplar genotypes^14,15^. In this study, for the first time in plants, we demonstrate that the systems of split–Kan^R^ and split–Hyg^R^ are effective for both *in planta* and plant tissue culture co-transformation in herbaceous and woody plants.

By dividing the larger cargoes across two T-DNAs, such systems enable the effective co-transformation of two separate binary vectors into a plant by *Agrobacterium*-mediated transformation. One constraint is that the insertion sites of the two T-DNAs are not controlled. Thus the two T-DNAs will exhibit Mendelian segregation, as observed in Fig. 2a. In fact, most frequently, the offspring of crossings between different parents are used to study the inheritance of mutant traits in *A. thaliana*^16^. For example, by mating the two single knockout mutants, constitutive double knockout *Arabidopsis* mutants lacking both DPE2 and PHS1 were produced^17^. Yuan et al. generated a *pp2ab’αβ* double mutant by crossing two homozygous single-insertion mutants, *pp2ab’α* and *pp2ab’β*^18^. To develop homozygous *drm1drm2* double mutant plants, Cao and Jacobsen crossed two isolated singe mutants *drm1* and *drm2*, in order to examine the function of the *DRM* genes^19^. In general, a heterozygous double mutant will be generated in the F1 generation, which is equivalent to the T1 generation of this study, and a homozygous double mutant will be achieved in the F2 generation, which is equivalent to the T2 generation of this study. Therefore, a homozygous double mutant created by split–Kan^R^ or split–Hyg^R^ system is expected to be achieved in T2 generation for plant species with sexual reproduction, e.g., *Arabidopsis*, rice, and tomato. In contrast, for plants relying on vegetative propagation, e.g., poplar and citrus, the phenotype of double mutant will be inherited consistently without phenotype segregation.

To easily identify transgenic events in plant transformation, we chose *RUBY* and *eYGFPuv* as the selectable reporters which are visible to the naked eye under white and UV light, respectively, without a need for cost- and labor-intensive characterization^11–13^. Indeed, the green fluorescence of plants expressing eYGFPuv can be observed consistently both in *Arabidopsis* and poplar. The red pigment of plants expressing RUBY, in contrast, was less consistent, particularly in poplar, where no typical RUBY phenotype was found in this study. To address this issue, a more reliable reporter such as *GUS* or *LUC* tends to be a better option to replace the RUBY reporter.

The advantages of these co-transformation methods can reduce valuable time spent on constructing complex or long T-DNA molecules in binary vectors and sequential transformations, thus improving the capabilities for pathway engineering and genetic improvement of polygenic traits. In addition, the current common practice of expressing multiple genes involves the repeated use of the same or similar promoters due to the limited number of available promoters^20^. Here, repetitive sequences within a plasmid can undergo intramolecular DNA recombination^21^. This scenario is avoided with the use of the split selectable marker system described here. The choice of delivering multiple gene expression cassettes containing multiple identical sequences with two transformation vectors should allow a drastic reduction in the frequency of plasmid DNA recombination. Finally, this technology potentially doubles the capacity of existing transformation systems for multi-gene engineering in plants.

## Methods

### Plant materials

*Arabidopsis* (*A. thaliana*) ecotype Columbia-0 (Col-0) and tobacco (*Nicotiana benthamiana*) were grown in controlled-climate chambers under fluorescent cold white light (100–150 µmol m^−2^ s^−1^), 16-h light/8-h dark photoperiod, 20–22 °C, and 60% humidity. In vitro-grown poplar ‘717’ (*Populus tremula* × *P. alba* clone INRA 717-1B4) plantlets were placed in a growth room with a photoperiod of 16-h light/8-h dark at 22 °C.

### Vector construction

To split *RUBY*, we first created a *RUBY*-minus vector lacking the gene *GT* by assembling PCR product 1 containing *CYP76AD1* and *DODA* and PCR product 2 containing *Arabidopsis* HSP18.2 terminator into a pGFPGUSplus vector^22^ via NEBuilder HiFi DNA Assembly (New England BioLabs). The pAXY0006 vector of split-*RUBY* was generated by assembling PCR products containing f1 fragment of gene *GT* (named GTf1) and NpuDnaE(N) into RUBY-minus vector via NEBuilder HiFi DNA Assembly. The pAXY0007 vector of split-RUBY was generated by assembling PCR products containing an f2 fragment of gene *GT* (named GTf2) and NpuDnaE(C) into pGFPGUSplus vector via NEBuilder HiFi DNA Assembly. To split Kan^R^ (i.e., *nptII*) and Hyg^R^ (i.e., *hpt*), gBlocks Gene Fragments containing either 5′-Kan^R^/Hyg^R^ and N-terminal of NpuDnaE or C-terminal of NpuDnaE and 3′-Kan^R^/Hyg^R^ were synthesized from Integrated DNA Technologies IDT. The pAXY0008/00010/00012/00014 vectors of split-Kan^R^**/**Hyg^R^ were generated by assembling PCR products containing F1/F3 fragments of Kan^R^/Hyg^R^ and NpuDnaE(N) into pGFPGUSplus vector via NEBuilder HiFi DNA Assembly. The pAXY0009/00011/00013/00015 vectors of split-Kan^R^**/**Hyg^R^ were generated by assembling PCR products containing F2/F4 fragment of Kan^R^/Hyg^R^ and NpuDnaE(C) into pGFPGUSplus vector via NEBuilder HiFi DNA Assembly. The coding sequences of inteins were codon optimized for *Arabidopsis* via the online codon optimization tool (ExpOptimizer) provided by NovoPro Bioscience (Shanghai, China). All vectors were verified by Sanger sequencing. Information for all primers, gBlocks, and plasmids used in this study is provided in Supplementary Data 1 and Supplementary Table 1.

### *Arabidopsis* stable transformation

The *A. tumefaciens* strain ‘GV3101’ was used for the transformation of *Arabidopsis* wild type ‘Col-0’ via the floral dip method as described previously^23^. For co-transformation, two *Agrobacterium* strains containing corresponding vectors (Supplementary Fig. 2), respectively, were cultured separately overnight in 100 mL LB liquid medium supplied with 50 mg/L Kanamycin and 50 mg/L Rifampicin. Two LB cultures were spun down at 4000–5000 rpm for 20 min and resuspended in 30 mL new LB liquid medium without antibiotics and mixed equally. Mixed LB culture was added into 120 mL dip solution containing 5% sucrose and 0.03% Silwet-L77. In general, 8–12 plants were used for each co-transformation.

### Poplar stable transformation

The *A. tumefaciens* strain ‘EHA105’ was used for the co-transformation of the poplar ‘717’ following a published method^24^. 50 mL LB culture for each *Agrobacterium* strain was prepared and spun down as described above. Two *Agrobacterium* pellets were resuspended equally in an MS induction medium containing 20 μM acetosyringone at an OD600 nm of 0.5–0.8 for each strain. Excised leaf disks from young leaves (~150) were soaked in *Agrobacterium* solution for 1 h, followed by multiple steps, including co-culture, washing, callus induction, shoot induction, shoot elongation, and root induction.

### Tobacco leaf infiltration

Infiltration of tobacco leaves was performed following a published method^24^. For co-infiltration, a 5 mL overnight culture of two *Agrobacterium* strains was spun down and resuspended equally in a resuspension solution containing 10 mM MgCl_2_, 10 mM MES-K (pH 5.6), and 100 μM acetosyringone at an OD600 nm of 0.5 for each strain.

### Genotyping

To genotype the resistant lines, leaves, approximately 0.5–1.0 cm, were collected from *Arabidopsis* and poplar ‘717’ and ground to a powder. Genomic DNA was isolated by a modified sodium dodecyl sulfate (SDS)-based DNA extraction method^25^. Forward primer 5′-CACGGCAACCTCAACG-3′ and reverse primer 5′-CTCGACACGTCTGTGGG-3′ were used for genotyping PCR of *eYGFPuv*. Forward primer 5′- CAGAGCTTGCGAGAAAGG-3′ and reverse primer 5′- GGCGGAGGTGAACTTGTAG-3′ were used for genotyping PCR of *RUBY*.

### Phenotyping

The fluorescence signals of eYGFPuv were visualized under a 365 nm wavelength UV light and imaged using an iPhone 11 as described by Yuan et al.^11^. The red pigment due to RUBY expression is visible by naked eyes without requiring any equipment^12^, and images were also taken using an iPhone 11.

### Protein extraction and Western blot

HEK 293 T cells were obtained from ATCC and maintained in a humidified atmosphere at 5% CO_2_ in Dulbecco’s Modified Eagle’s (DMEM) complete medium (Corning) supplemented with 10% fetal bovine serum (FBS; Seradigm) in 37 °C. Plasmid transfections were done with TransIT-LT1 (Mirus Bio) per the manufacturer’s instructions. Briefly, cell extracts were generated on ice in EBC buffer, 50 mM Tris (pH 8.0), 120 mM NaCl, 0.5% NP40, 1 mM DTT, and protease and phosphatase inhibitors tablets (Thermo Fisher Scientific). Extracted proteins were quantified using the PierceTM BCA Protein assay kit (Thermo Fisher). Proteins were separated by SDS acrylamide gel electrophoresis and transferred to IMMOBILON-FL 26 PVDF membrane (Millipore) probed with the indicated antibodies and visualized either by chemiluminescence (according to the manufacturer’s instructions) or using a LiCor Odyssey infrared imaging system. Western blot was conducted as described previously^10^. Primary antibodies used for western blot are HA (Cat# 902302; 1:1000 dilution) from Biolegend and M2 FLAG (Cat# F1804; 1:1000 dilution) antibody from Sigma. Secondary antibodies used are IRDye 800CW Goat anti-Mouse IgG Secondary Antibody (LiCor) and IRDye 680RD Goat anti-Rabbit IgG Secondary Antibody (LiCor). The study used a primary antibody against β-actin (Cat# A1978 from Sigma) for internal protein control.

### Statistics and reproducibility

The experiment of tobacco leaf infiltration was performed two times and with two replicates each time. The stable transformation of both *Arabidopsis* and poplar was conducted two times. For each *Arabidopsis* transformation, a minimum of eight individual plants were utilized. Similarly, at least 150 explants were employed for each poplar transformation. A minimum of five transgenic events were chosen for PCR and phenotype analysis in *Arabidopsis*, while fifteen transgenic events were selected for the same analysis in Poplar. The number of replicates was described in each figure and figure legend.

### Reporting summary

Further information on research design is available in the Nature Portfolio Reporting Summary linked to this article.

## Supplementary information

Supplementary Information

Description of Additional Supplementary Files

Supplementary Data 1

Reporting Summary

## Data Availability

The plasmids will be available at Addgene. All other data are available from the corresponding author upon reasonable request.
