# Supplementary material for: Split selectable marker systems utilizing inteins facilitate gene stacking in plants
Source: Commun Biol. 2023 May 26;6:567. doi: 10.1038/s42003-023-04950-8 (PMC10219933; doi:10.1038/s42003-023-04950-8)
Supplement: Supplementary file 1 — Supplementary Information [file 42003_2023_4950_MOESM1_ESM.pdf]

Split selectable marker systems utilizing inteins facilitate gene stacking in plants

Guoliang Yuan[1,2,3], Haiwei Lu[1,4], Kuntal De[1], Md Mahmudul Hassan[1,2,5], Yang Liu[1], Md. Torikul Islam[1], Wellington Muchero[1,2], Gerald A. Tuskan[1,2*], Xiaohan Yang[1,2*]

[1]Biosciences Division, Oak Ridge National Laboratory, Oak Ridge TN 37831, USA
[2]The Center for Bioenergy Innovation, Oak Ridge National Laboratory, Oak Ridge, TN 37831, USA
[3]Chemical and Biological Process Development Group, Pacific Northwest National Laboratory, 902 Battelle Boulevard, Richland, WA 99352, USA
[4]Department of Academic Education, Central Community College – Hastings, Hastings, NE 68902, USA
[5]Department of Genetics and Plant Breeding, Patuakhali Science and Technology University, Dumki, Patuakhali-8602, Bangladesh

*Corresponding authors: Gerald A. Tuskan (tuskanga@ornl.gov); Xiaohan Yang (yangx@ornl.gov)

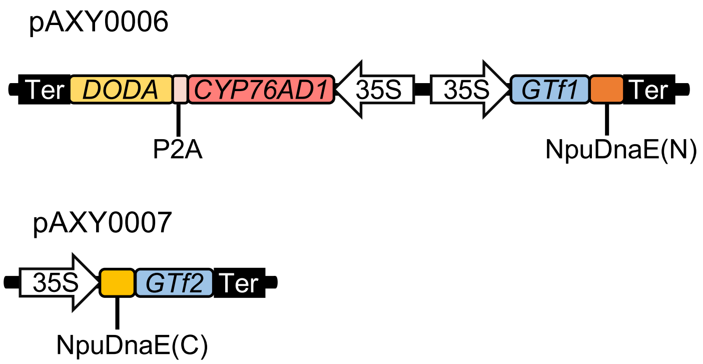

Supplementary Figure 1. Illustration of split-Kan^R and -Hyg^R vectors.

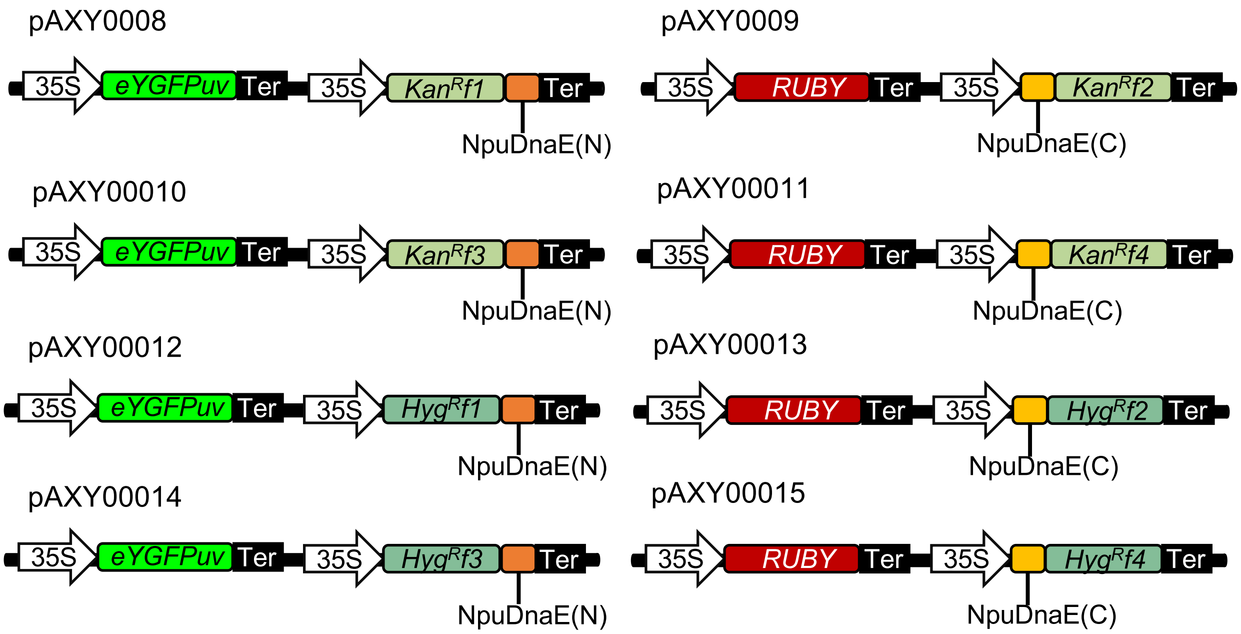

Supplementary Figure 2. Phenotyping of Kanamycin-resistant and Hygromycin-resisted T2 transformants.

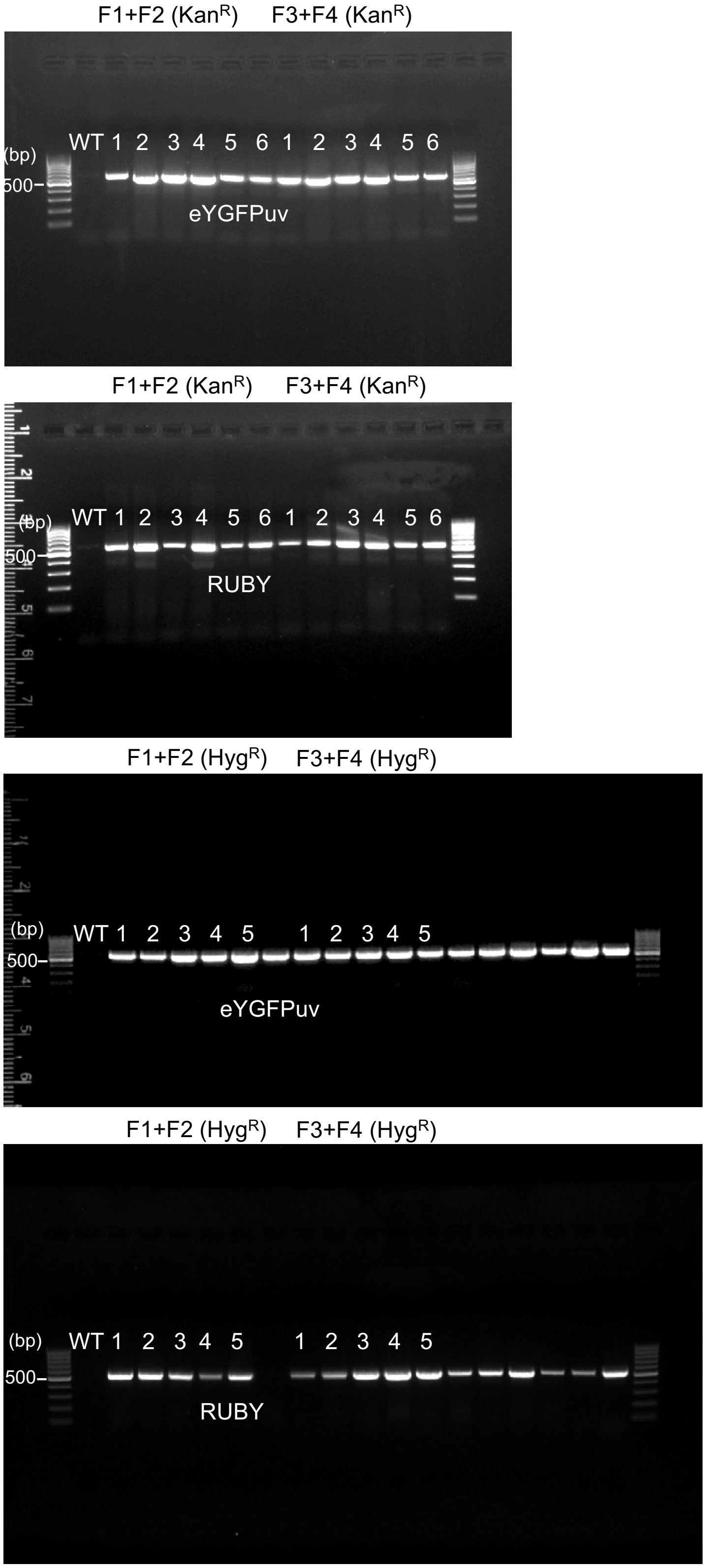

Supplementary Figure 3. Genotyping of T1 transformants using primers of *eYGFPuv* and *RUBY*, respectively (Uncropped and unedited gel images of Figure 1j).

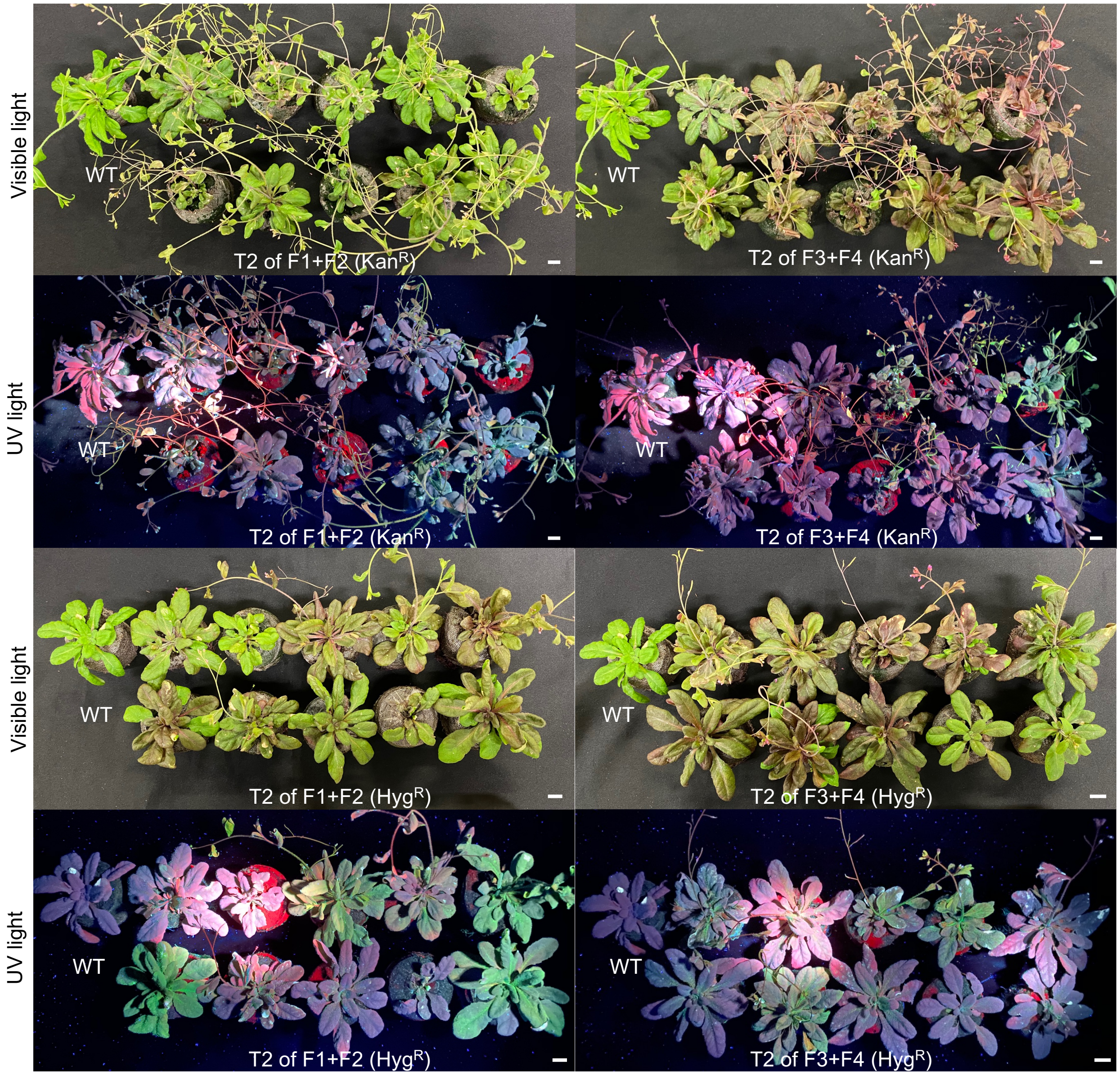

Supplementary Figure 4. Phenotyping of Kanamycin-resistant and Hygromycin-resisted T2 transformants. Scale bar, 1 cm.

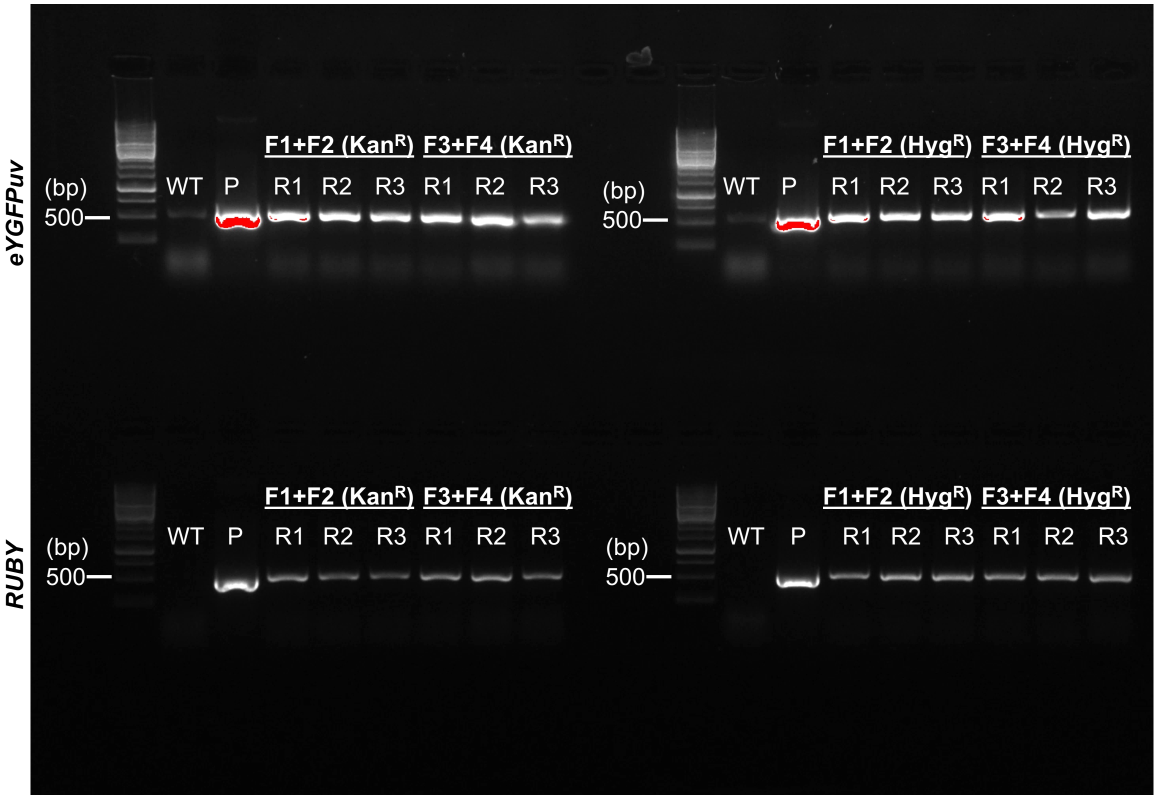

Supplementary Figure 5. Genotyping of T2 transformants using primers of *eYGFPuv* and *RUBY*, respectively.
The genomic DNA of wild type (WT) *Arabidopsis* was used as a negative control while eYGFPuv and RUBY plasmids were used as positive control for *eYGFPuv* and *RUBY*, respectively. R indicates biological replicates.

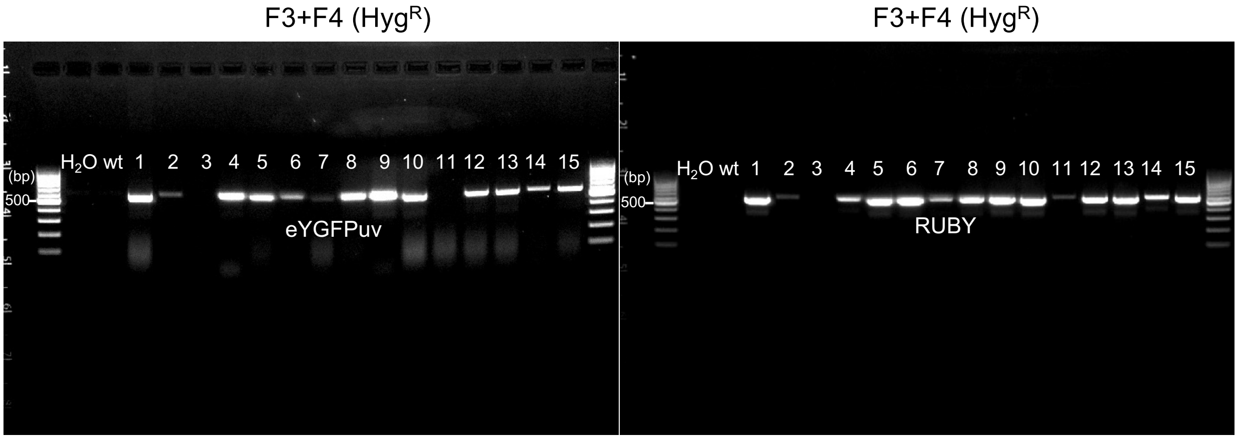

Supplementary Figure 6. Genotyping of transgenic poplar events using primers of *eYGFPuv* and *RUBY*, respectively (Uncropped and unedited gel images of Figure 3b).

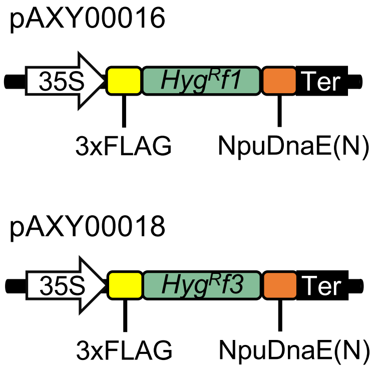

Supplementary Figure 7. Illustration of split-Hyg^R vectors with FLAG or HA epitope.

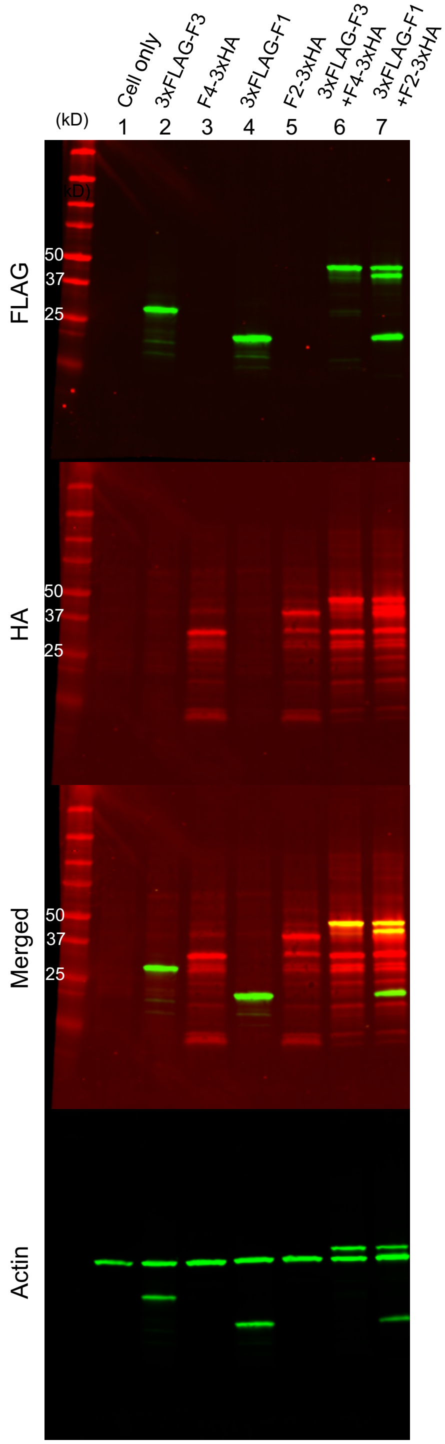

Supplementary Figure 8. Western blot analysis of trans-splicing of the HygR protein (Uncropped and unedited blot images of Figure 4b).

Western blot was performed with the proteins extracted from human kidney cells, which were either transfected with the plasmids containing one of fragment F1 to F4, respectively, or co-transfected with F1+F2 containing plasmids and F3+F4 containing plasmids. Red, green, and yellow bands indicate FLAG, HA, and merged bands, respectively. Actin serves as the equal-loading control.

Supplementary Table 1. Plasmids used in this study.

Plasmid #	pXYA#	Plasmid Name
1	pAXY0006	pGlucos(1-231)-NpuDnaE(N)
2	pAXY0007	pNpuDnaE(C)-Glucos(232-500)
3	pAXY0008	pKan^R(1-131)-NpuDnaE(N)_eYGFPuv.
4	pAXY0009	pNpuDnaE(C)_Kan^R(132-265)_RUBY.
5	pAXY0010	pKan^R(1-192)-NpuDnaE(N)_eYGFPuv.
6	pAXY0011	pNpuDnaE(C)_Kan^R(193-265)_RUBY.
7	pAXY0012	pHyg^R(1-52)-NpuDnaE(N)_eYGFPuv
8	pAXY0013	pNpuDnaE(C)_Hyg^R(53-341)_RUBY
9	pAXY0014	pHyg^R(1-89)-NpuDnaE(N)_eYGFPuv
10	pAXY0015	pNpuDnaE(C)_Hyg^R(90-341)_RUBY
11	pAXY0016	p3flag_Hyg^R(1-89)-NpuDnaE(N)
12	pAXY0017	pNpuDnaE(C)_Hyg^R(90-341)_3HA
13	pAXY0018	p3flag_Hyg^R(1-52)-NpuDnaE(N)
14	pAXY0019	pNpuDnaE(C)_Hyg^R(53-341)_3HA
