# Supplementary material for: Split selectable marker systems utilizing inteins facilitate gene stacking in plants
Source: Commun Biol. 2023 May 26;6:567. doi: 10.1038/s42003-023-04950-8 (PMC10219933; doi:10.1038/s42003-023-04950-8)
Supplement: Supplementary file 2 — Description of Additional Supplementary Files [file 42003_2023_4950_MOESM2_ESM.pdf]

Description of Additional Supplementary Files

File name: Supplementary Data 1

Description: Information for all primers, gBlocks and plasmids used in this study.
